# An Advanced IVB Lung Adenocarcinoma Patient With KRAS Mutations, Benefited From Camrelizumab Combined With Anti‐Angiogenic Agents for Therapy: A Case Report

**DOI:** 10.1002/cnr2.70186

**Published:** 2025-06-09

**Authors:** Li Wang, Jiaqi Wu, Ping Shao, Wuping Bao, Lin Mao, Zhendong Pan, Aihua Bao, Min Zhang, Zhenghua Wu, Guorong Fan

**Affiliations:** ^1^ School of Pharmacy Shanghai Jiao Tong University Shanghai China; ^2^ Department of Clinical Pharmacy Shanghai General Hospital, Shanghai Jiao Tong University, School of Medicine Shanghai China; ^3^ Department of Respiratory Medicine Shanghai General Hospital, Shanghai Jiao Tong University School of Medicine Shanghai China; ^4^ Department of Pharmacy EYE & ENT Hospital of Fudan University Shanghai China

**Keywords:** anti‐angiogenic agents, camrelizumab, case report, KRAS mutations, lung adenocarcinoma

## Abstract

**Background:**

Although the presence of Kirsten murine sarcoma virus (KRAS) mutations predicts a failure of non‐small cell carcinoma (NSCLC) patients to benefit from epidermal growth factor receptor (EGFR)—tyrosine kinase inhibitor (TKI) therapy it may be more sensitive to programmed combination therapy of programmed death 1 (PD‐1)/programmed death ligand 1 (PD‐L1) inhibitors + anti‐angiogenesis. Recent treatment guidelines and clinical studies related to adenocarcinoma in NSCLC have indicated that in patients with inoperable stage IV lung adenocarcinoma, immune checkpoint inhibitors in combination with anti‐angiogenic drugs may exert a synergistic effect and significantly improve the efficacy of near‐term treatment, but quantification and long‐term follow‐up of specific clinical indicators are still lacking. No previous cases of long‐term good results with camrelizumab combined with anti‐angiogenic agents for KRAS‐mutated NSCLC have been described.

**Case:**

This manuscript reports a case of a patient with advanced NSCLC with pleural effusion and KRAS mutations treated poorly with conventional chemotherapy who had long‐term (more than 18 months) benefit with immunotherapy combined with an anti‐angiogenic inhibitor in Shanghai General Hospital. In this case, pharmaceutical care of the patient was carried out through therapeutic drug adjustment, compliance, efficacy assessment, and safety evaluation to provide a reference for improving the efficacy and safety of drug therapy in clinical practice. As of the last follow‐up date (December 2023), overall survival was 27 months, and the patient is currently in good general condition with no significant complaints of discomfort.

**Conclusion:**

ICLs in combination with antiangiogenic therapy may be a therapeutic option for patients with KRAS mutations in advanced non‐small cell lung cancer with good persistence.

**Abbreviations**
AFP: Alpha‐fetoprotein
ALK: Anaplastic lymphoma kinase
ALT: Alanine transaminase
AP: Chemotherapy regimen of pemetrexed in combination with cisplatin
AST: Aspartate Transaminase
CA125: Carbohydrate antigen 125
CA153: Carbohydrate antigen 153
CA199: Carbohydrate antigen 199
CA724: Carbohydrate antigen724
CEA: Carcinoembryonic antigen
CYFRA21‐1: cytokeratin 19 fragments
EGFR: Epidermal growth factor receptor
IL‐6: Interleukin‐6
Ki67: A protein encoded in humans by the MKI67 gene (an antigen recognized by the monoclonal antibody Ki‐67)
KRAS: Kirsten rat sarcoma viral oncogene homolog
LAC: lung adenocarcinoma
MET: Cellular‐mesenchymal to epithelial transition factor
MPE: Malignant pleural effusion
MPMN: Multiple Primary Malignant Neoplasm (oncology)
NSCLC: Non‐small‐cell‐lung‐cancer
NSE: Neuron‐specific enolase
PD‐1/PD‐L1: Programmed cell death 1 ligand 1
Pro‐GRP: Gastrin‐releasing peptide precursor
ROS1: ROS proto‐oncogene 1, receptor tyrosine kinase
SCC: Squamous cell carcinoma‐associated antigen
SCLC: Small‐cell‐lung‐cancer
TC: Total cholesterol
TG: Triglycerides
TKI: Tyrosine kinase inhibitors
TP: Combination of paclitaxel‐based chemotherapeutic agents with platinum‐based agents
VEGF: Vascular endothelial growth factor

## 1 Introduction

### 1.1 Background

Lung cancer is one of the most commonly diagnosed cancers and the leading cause of cancer‐related deaths worldwide [1]. Lung cancer, also referred to as primary bronchial lung cancer, is divided into small cell lung carcinoma (SCLC) and non‐small cell lung carcinoma (NSCLC), of which NSCLC accounts for approximately 85% of all lung cancer types. Adenocarcinoma, a type of NSCLC, is the least associated with smoking and accounts for 40% of primary lung tumors [2]. It is often located in the peripheral part of the lung, which also involves the pleura and forms an associated scarring circle and pleural effusion, and has a poor prognosis. Malignant pleural effusion (MPE) frequently complicates advanced lung adenocarcinoma (LAC) and is linked to diminished life expectancy [3]. The expression of vascular endothelial growth factor, a factor that has recently been shown to play an important role in the formation of malignant pleural effusions, has increased significantly, leading to the emergence of angiogenesis inhibitors as a key approach to control tumor progression [4].

### 1.2 Rationale and Knowledge Gap

The efficacy of chemotherapy for advanced NSCLC has reached a bottleneck, and molecular targeted therapy is due to its targeting of the altered characteristics of tumor cells, thereby demonstrating enhanced anti‐tumor activity while minimizing toxic side effects on normal cells. This kind of targeted therapeutic approach has pointed out a new direction for tumor treatment. According to the role of the target and nature of the drug, the main molecular targeted therapy drugs include the following categories: (1) Small molecule epidermal growth factor receptor (EGFR) tyrosine kinase inhibitors, such as gefitinib, erlotinib; (2) Anti‐EGFR monoclonal antibodies, such as cetuximab; (3) Anti‐HER‐2 monoclonal antibody, for example, trastuzumab; (4) VEGF receptor inhibitors, for example, bevacizumab, and so forth. The formation of new blood vessels represents one of the principal mechanisms underlying tumor growth and resistance; it also facilitates tumor cell migration and distant metastasis. Bevacizumab is a humanized monoclonal antibody that selectively binds to vascular endothelial growth factor (VEGF), thereby inhibiting the formation of tumor neovascularization. The development of new blood vessels is one of the main mechanisms of tumor growth and resistance, and it also provides a pathway for tumor cell migration and distant metastasis. In contrast to conventional cancer therapies, immune checkpoint inhibitors do not directly target cancer cells; instead, they mobilize the immune system to re‐recognize cancer cells that have evaded surveillance, thus restoring its functionality. Camrelizumab is a novel human immunoglobulin G4 (IgG4)‐based monoclonal antibody (mAb) that blocks pathways associated with PD‐1 and PD‐L1. This action disrupts T‐cell immunosuppression and enhances T‐cell‐mediated cytotoxicity against tumor cells. The 2020 guidelines on anti‐angiogenesis in advanced NSCLC suggest that platinum‐containing chemotherapy combined with anti‐angiogenic therapy or immune checkpoint inhibitor immunotherapy is recommended as first‐line treatment for patients with advanced non‐squamous NSCLC who are negative for driver genes [5]. In addition to medication selection, the treatment efficacy and five‐year survival rates for lung cancer are closely linked to early diagnosis and timely intervention [6]. Tumor markers demonstrate higher sensitivity in diagnosing tumors; furthermore, serum specimens are more readily accessible and less invasive than other diagnostic approaches. Imaging studies and histocytological examinations serve as primary diagnostic tools for diagnosing lung cancer, but both methods exhibit limitations such as low sensitivity, specificity, and invasiveness. Tumor markers have a higher sensitivity for tumor diagnosis; in addition, serum specimens are more easily available and less invasive to the body. Previous studies have confirmed that serum levels of tumor markers such as neuron‐specific enolase (NSE), carcinoembryonic antigen (CEA), cytokeratin 19 fragments (CYFRA21‐1), carbohydrate antigen 125 (CA125)and carbohydrate antigen 153 (CA153) are significantly higher in lung cancer patients than in patients with benign lung lesions and healthy individuals, and the levels are significantly higher in patients with postoperative recurrence than in patients without recurrence [7, 8]. This suggests that monitoring serum tumor markers has an important impact on the prognosis of lung cancer patients.

Recent treatment guidelines have recommended the combination of immune checkpoint inhibitors and anti‐angiogenic drugs for the treatment of stage IV lung adenocarcinoma [5], and clinical trials have confirmed the efficacy of this regimen, camrelizumab combined with bevacizumab. However, there remain significant knowledge gaps regarding optimal methods for monitoring and evaluating the long‐term effectiveness and safety of this therapeutic approach. Although current monotherapy with immune checkpoint inhibitors (ICIs) has a manageable safety profile, their efficacy is greatly limited by low response rates. Therefore, scientists have sought various combination therapies to enhance response rates to ICIs. Combination regimens have primarily shown better in vivo clinical activity compared to monotherapy; however, the management of adverse effects is also more problematic. There is still a lack of long‐term follow‐up evidence to confirm the durable response and survival benefits from combination therapies of ICIs combined with anti‐angiogenesis.

### 1.3 Objective

Based on the above background, this case reports information on the adjustment from camrelizumab in combination with recombinant human vascular endothelial inhibitor to camrelizumab in combination with bevacizumab for the treatment of stage IV lung adenocarcinoma with long‐term follow‐up in terms of efficacy and safety. To provide additional evidence of durable responses from combination therapy combining ICLs with anti‐angiogenesis. The detailed report is below.

## 2 Case Presentation

**FIGURE 1:**
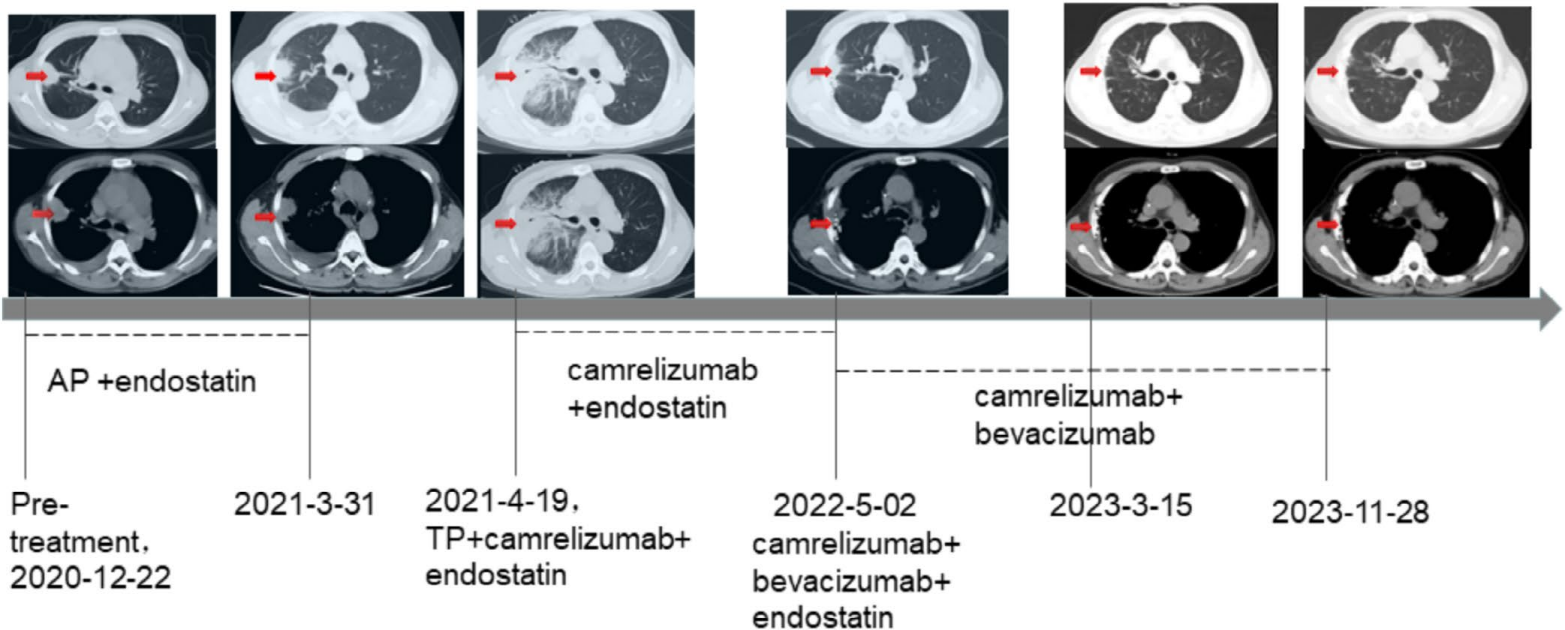
Details of the pathological images and treatments during the course of the disease. Red arrows indicate tumor lesions or nodules.

In December 2020, a male patient presented was admitted to the Respiratory Department of the Shanghai General Hospital with symptoms of cough, sputum, and chest tightness that persisted for a month and worsened over one week. Ultrasound showed a large pericardial effusion, hypoechoic nodules measuring approximately 31 × 40 mm (Figure 1) and a small amount of pleural effusion, and a diagnosis of NSCLC (adenocarcinoma of the lung) with tumor stage IVB (T3N3MIC) was considered after a puncture biopsy of the corresponding site. The excisional biopsy samples from the patient's lung tissues were treated with neutral formalin and subsequently subjected to somatic mutation analysis. This included an assessment of all exons across 11 genes along with their mutated forms—such as point mutations, small insertion‐deletions, copy number variations, and fusions—in six rearrangement genes. The reference genome of was GRCh37/hg19 to predict the patient's susceptibility to targeted drugs. Genomic testing based on next‐generation sequencing showed that the patient had a p. Q61H mutation in p. Q61X of KRAS and MET amplification (more details in Table 1).

**TABLE 1:** Patient genomic variations information.

Gene name	Area covered	Mutation type	Testing results
EGFR (Epidermal growth factor receptor)	All exon	p. L858R	No mutation
		p. G719X	No mutation
		p. S7681	No mutation
		p. T790M	No mutation
		p. C797S	No mutation
		p. L718Q	No mutation
		Other types	No mutation
ALK (Anaplastic lymphoma kinase)	All exon and intron18–19	Genetic rearrangements	No mutation
		Other types	No mutation
ROS1 (ROS proto‐oncogene 1, receptor tyrosine kinase)	All exon and intron 31–35	Genetic rearrangements	No mutation
		Other types	No mutation
MET (Cellular‐mesenchymal to epithelial transition factor)	All exon	Copy number amplification	Amplification
		Genetic rearrangements	No mutation
		Other types	No mutation
KRAS (Kirsten rat sarcoma viral oncogene homolog. EGFR: Epidermal growth factor receptor)	All exon	p. G12X	No mutation
		p. G13X	No mutation
		p. Q61X	p. Q61H
		p. K117N	No mutation
		p. A146X	No mutation
		Other types	No mutation

The patient was in good health, denied any history of chronic medical conditions such as hypertension, diabetes, and family history of tumors, and admitted to a history of smoking, 20 cigarettes/day, for over 30 years. In March 2021, when the patient was scheduled for his fifth chemotherapy treatment, a chest CT revealed an increase in pleural thickening, an increase in peripheral inflammation, and a significant increase in pleural effusion after 3 months of using cisplatin (100 mg) and pemetrexed (800 mg) (AP scheme) in combination with endothelial inhibitors. The diameter of the tumor was 39 mm × 36 mm (Figure 1). On 20 April 2021, the patient was admitted to the hospital with dyspnea and was diagnosed with malignant pleural effusion; the treatment regimen was adjusted to the combined treatment of Cisplatin (100 mg) and Docetaxel (120 mg) (TP scheme) in combination with camrelizumab (200 mg) and endostatin (210 mg) was administered on 2 September 2021, reconsidering the progression of the disease, the combined treatment of camrelizumab (200 mg) and endostatin (210 mg) was administered; during this period, the patient underwent proton heavy ion therapy 6 times at external hospitals (details not available). On 20 March 2022, while continuing treatment with camrelizumab, an ultrasound‐guided biopsy of the thyroid gland and lymph nodes was performed, and papillary thyroid cancer was identified. The patient's condition was discussed as multiple primary malignancies (MPMN) [9], which refers to the simultaneous or sequential occurrence of 2 or more primary malignancies in the same body organ, the mechanism of which is currently unknown and perhaps related to the patient's reduced ability to monitor immune function to clear the mutated cells. Given the critical nature of the patient's primary disease at this time, neither the patient nor the physician considered temporary management in relation to papillary thyroid cancer. Serum lung tumor markers were further controlled with targeted therapy. Changes in lung tumor serology during respiratory chemotherapy are shown in Table 2 and imaging reports are shown in Figure 1.

**TABLE 2:** Serological indicators of lung tumor during chemotherapy.

Serological markers of tumor (normal range)	Pre‐treatment	AP (pemetrexed cisplatin) + endostatin	TP (paclitaxel platinum) + camrelizumab+endostatin	Camrelizumab+endostatin			Camrelizumab + bevacizumaab + endostatin	Camrelizumab + bevacizumab		
	2020/12/22	2021/3/31	2021‐04‐20 (treatment for malignant pleural effusion)	2021/9/2	2021/12/15	2022/3/30	2022/5/2	2022/7/15	2023/3/15	2023/11/28
IL‐6, pg/ml	157.1	—	—	—	—	—	—	—	—	—
SCC (Squamous cell carcinoma‐associated antigen), ng/ml (0–1.5 pg/mL)	2.1	0.6	1.2	1.2	4	1	1.3	0.8	1.3	1.6
Pro‐GRP (Gastrin‐releasing peptide precursor), pg/ml (25.3–77.8 pg/mL)	43.94	26.43	28	37.1	48.34	36.8	42.69	31.8	32.00	34.05
NSE (Neuron‐specific enolase), ng/ml (0–16.3 pg/mL)	42.32	15.52	29.88	9.43	12.79	12.5	11.56	11.45	8.93	13.13
CYFRA211, ng/ml	7.67	3.42	4.08	2.29	3.26	2.25	1.61	2.8	2.59	17.6
CA199, U/mL (0–25 U/mL)	17.7	20.9	20.7	14.4	16.4	15.1	13	17.4	13.6	10.1
CEA (Carcinoembryonic antigen), ng/ml (0–5 ng/mL)	13.43	43.08	33.68	9.3	6.85	14.31	12.83	7.97	10.82	3.82
AFP(Alpha‐fetoprotein), ng/ml (0–9 ng/mL)	1.96	6.25	11.4	3.06	3.89	3.32	3.04	3.2	3.05	3.02
CA153, U/mL (0–14 U/mL)	—	34.5	42.2	11.4	8.1	20.8	22.2	12.9	12.60	—
CA125, U/mL (0–35 U/mL)	—	347.3	604.7	51.6	7.4	129.3	134.7	20.7	31.7	—

The patient's liver and kidney function were monitored regularly during the drug administration, and no significant hepatic or renal adverse effects were observed in this patient. Since the patient had a fatty liver, the patient's lipid profile and liver function were monitored, as shown in Table 3. After 12 cycles of immunotherapy, the patient's dosing regimen was adjusted from camrelizumab (200 mg) in combination with endonuclease (210 mg) to a combination of camrelizumab (200 mg) and bevacizumab (900 mg) with a higher recommendation grade according to the relevant clinical treatment guidelines [5]. This patient's dosing regimen was all based on a 21‐day dosing cycle, with all antineoplastic drugs administered intravenously, except for recombinant human vascular endothelial inhibitors, which were administered intra‐pump. Interestingly, within 18 months of immunotherapy, we not only found that the patient's pleural effusion disappeared but also that the levels of several tumor markers tested fell from above normal to the normal range. In addition, the primary tumor in the lung did not progress. As of the last follow‐up date (December 2023), the overall survival was 27 months.

**TABLE 3:** Monitoring of blood lipids and transaminases during drug administration.

Monitoring Indicators (normal range)	Camrelizumab+endostatin			Camrelizumab + bevacizumaab + endostatin	Camrelizumab+bevacizumab		
	2021/9/2	2021/12/15	2022/3/30	2022/5/2	2022/7/15	2022/3/15	2023/11/28
TG(Triglycerides) mmol/L, (0–1.7 mmol/L)	0.95	3.1	2.87	4.35	2.64	3.88	4.52
TC(Total Cholesterol) mmol/L, (2.85–5.69 mmol/L)	6.15	5.31	5.37	6.06	5.08	5.15	5.08
AST/ALT (Aspartate Transaminase/Alanine transaminase) (0.8–1.5 mmol/L)	1.8	2.1	1.9	2.7	1.8	1.95	2.12

The patient went on to pursue a successful career as a staff member in China. After one year of maintenance immunotherapy, he reported no discomfort, which has significantly improved the quality of life. The regimen was maintained as the patient remained symptom‐free, and the results of the review were satisfactory. According to NSCLC guidelines, bevacizumab in combination with an immune checkpoint inhibitor significantly prolongs progression‐free survival and/or overall survival in this disease, and the patient is currently in good health [10]. As things stand, his risk of lung adenocarcinoma recurrence is low, which is reassuring, especially since his career will require a long‐term deployment with limited medical resources. However, if any symptoms of lung adenocarcinoma develop during deployment, then further medical intervention will be necessary.

## 3 Discussion

### 3.1 Key Findings

In this case, the patient's entire course of treatment covered a comprehensive range of therapeutic measures, with the effects of chemotherapy alone, single PD‐1 antibody therapy combined with Endostatin, and dual anti‐targeted therapy all being demonstrated. In particular, dual anti‐targeted therapy with camrelizumab combined with bevacizumab has already been observed to have better efficacy in 18 months of treatment with no significant adverse effects, providing a new referenceable treatment option for patients with advanced NSCLC with KRAS mutation. Similar reports have been limited to the efficacy of ICL combined with bevacizumab in patients with advanced cholangiocarcinoma, hepatocellular carcinoma, and cervical carcinoma, mostly focusing on the inhibition of PD‐1 expression, ALK positivity, and EGFR mutation, and so forth [11, 12, 13, 14]. However, the effect is not always in line with the expectations of medical personnel and patients, and patients experience different adverse effects in the late stage [12, 14]. The main outcome goals of similar reports were tumor size on imaging and patient progression‐free survival (PFS), while further details on the actual efficacy and evaluation of the regimen are lacking [15].

### 3.2 Explanations of Findings

The occurrence, development, and prognosis of lung cancer are closely related to the immune function status of the body and the tumor microenvironment (TME) [16, 17, 18]. The TME encompasses the environment surrounding a tumor and includes the surrounding blood vessels, immune cells, fibroblasts, signaling molecules, and extracellular matrix (ECM). Tumors can modulate their microenvironment by releasing extracellular signals, promoting tumor angiogenesis, and inducing peripheral immune tolerance. This modulation subsequently regulates the growth and evolution of the cancer cells themselves. In central or peripheral neurons, cell proliferation is accelerated in response to cancerous transformation and is accompanied by enhanced glycolysis and elevated NSE expression. Therefore, in SCLC and NSCLC with neuroendocrine characteristics, the elevated NSE level can be monitored for its efficacy and recurrence monitoring [19]. Gastrin‐releasing peptide precursor (Pro‐GRP) is closely related to gastrin‐releasing peptide (GRP). As the disease of NSCLC patients develops, the number of tumor cells increases, and the synthesis and secretion of GRP increase; the expression of serum Pro‐GRP is also upregulated. When GRP interacts with its receptor, the biological behaviors of tumor cells are altered, and their proliferation, invasion, and metastasis are intensified, leading to further deterioration of the condition of NSCLC patients, and the prognosis of their survival is greatly deteriorated [20]. Anti‐PD‐1 or anti‐PD‐L1 therapy may enhance the sensitivity and prolong the efficacy of anti‐angiogenic treatments. Conversely, anti‐angiogenic therapy can improve the effectiveness of anti‐PD‐L1 therapy by facilitating vascular changes that promote increased infiltration and activity of cytotoxic T cells, as well as aiding in tumor cell destruction. When high endothelial cell microvessel formation is present within tumors, the combination of anti‐angiogenic inhibitors with checkpoint inhibitors yields a more sustained therapeutic effect. Meanwhile, combined monitoring of tumor markers such as serum CEA, NSE, CYFRA21‐1, and Pro‐GRP is important in the prognostic assessment of lung cancer patients [19, 21].

### 3.3 Strengths and Limitations

NSCLC accounts for more than 80% of lung cancers, the vast majority of which were found to be in advanced inoperable stages. Research indicates that based on East Asian NSCLC patients, immunotherapy targeting PD‐1/PD‐L1 exhibits suboptimal responses in EGFR mutant and ALK‐rearranged tumors while demonstrating improved responses in KRAS mutant tumors. This discrepancy may be attributed to KRAS mutations driving an inflammatory phenotype associated with adaptive immune resistance [22]. In contrast, bevacizumab in combination with a PD‐1 antibody improves the abnormal tumor vascularity and enhances the cytotoxicity and infiltration of T lymphocytes leading to a better response to immunotherapy [23]. These findings deserve further validation in prospective studies. The combination of immune checkpoint inhibitors with anti‐angiogenesis has previously demonstrated efficacy in the treatment of advanced NSCLC but has focused on the macro aspects of overall survival, progression‐free survival, grade 3 to 5 treatment‐related adverse events, and health‐related quality of life [24, 25, 26]. However, some practice‐related questions remain unanswered, such as the optimal therapeutic strategy, the role of different biomarkers in treatment selection, and the efficacy of immunotherapy based on specific clinical features. However, only a small proportion of patients may benefit from treatment with PD‐1/PD‐L1 antibodies alone, which may be related to the complexity and dynamics of the tumor microenvironment. While previous reports on Endostatin for the treatment of lung adenocarcinoma combined with malignant pleural fluid have mostly been in combination with AP chemotherapy regimens or TKI, this case explored the clinical efficacy of PD‐L1 inhibitors in combination with endostatin and dual anti‐targeted therapy. In this case, the patient developed more severe malignant pleural fluid after the conventional 5 cycles of AP regimen chemotherapy, so the treatment regimen was adjusted to endostatin and camrelizumab, which were more effective. After more than a year of maintenance treatment with a PD‐1 inhibitor in combination with endostatin (during which proton heavy ion therapy was administered to kill solid tumor lesions), the patient's condition stabilized, and he was now treated with the newly recommended and clinically proven dual‐target therapy regimen for further treatment. After 19 cycles of dual anti‐targeted therapy, serum lung tumor markers were further controlled. However, the efficacy of this immunotherapy regimen was not well documented in terms of imaging reports as the patient had undergone concurrent proton‐heavy ion therapy to eliminate the tumor lesions.

### 3.4 Implications and Actions Needed

Patients were monitored for potential adverse reactions under this treatment regimen by regular routine blood, blood biochemistry, and urinary routine examinations. The patient's renal function was not significantly affected, but the transaminase ratio was high and basically maintained in a stable state. The patient's lipid level was also within the range of concern due to the combination of fatty liver. It is noteworthy that the patient did not take appropriate lipid‐lowering drugs during our treatment; nevertheless, we cannot exclude the possibility that the patient had related drugs outside the hospital. During the transition treatment (camrelizumab + bevacizumab + endostatin), a significant increase in both triglyceride levels, total cholesterol levels, and transaminase ratios was found in this patient, suggesting that the combination of these three drugs may have had some effect on liver function. A combined analysis of efficacy and adverse effect indicators throughout the treatment period revealed that camrelizumab in combination with bevacizumab had significant efficacy and no significant adverse effects. A study of advanced non‐small cell lung cancer found that the presence of KRAS mutations (G12X, codon 13 and Q61H) was associated with better survival in ≥ 50% of PD‐L1 patients treated with immune checkpoint inhibitor monotherapy, while there was no significant survival difference in patients treated with chemoimmunotherapy [27]. Unfortunately, data on the classification of KRAS mutation types have not been reported. This may be one of the reasons for the better treatment outcome for the patient in this case. Further clinical studies are necessary to confirm whether all patients with non‐small cell lung cancer harboring KRAS p. Q61H can be effectively treated using this regimen to achieve significant efficacy.

## 4 Conclusions

This case reports a patient with stage IV lung adenocarcinoma treated with a combination of an antiangiogenic inhibitor and an immune checkpoint inhibitor who switched from a combination of camrelizumab and endonuclease to a combination of camrelizumab and bevacizumab with a sustained benefit over 18 months, suggesting that combination immunotherapy may be a treatment option for patients with KRAS mutations in advanced non‐small cell lung cancer. Second, the combination of anti‐angiogenesis inhibitors and immunotherapy may improve efficacy and have good persistence. Despite encouraging results from studies evaluating the safety and efficacy of this combination therapy, there is currently no head‐to‐head trial evidence comparing the therapeutic efficacy of ICIs with other therapies such as angiogenesis inhibitors. There is also a need to advance effective immunosurveillance techniques that are closely linked to clinical endpoints. Significant affinities may help to identify favorable immune biomarkers that can then be validated in larger patient cohorts.

## Author Contributions

Conception and design: Li Wang, Guorong Fan. Administrative support: Min Zhang. Provision of study materials for patients: Wuping Bao, Aihua Bao, Ping Shao. Collection and assembly of data: Jiaqi Wu, Lin Mao, Zhendong Pan. Data analysis and interpretation: Li Wang, Zhenghua Wu. Manuscript writing: Li Wang. Final approval of manuscript: All authors.

## Consent

Written informed consent was obtained from the patient for publication of this report.

## Conflicts of Interest

The authors declare no conflicts of interest.

## Data Availability

The data that support the findings of this study are available on request from the corresponding author. The data are not publicly available due to privacy or ethical restrictions.
